# A Novel Polytetrahydrofuran-Based Shape Memory Polyurethane Enhanced by Polyglycolide-Block-Polytetrahydrofuran-Block-Polyglycolide Copolymer

**DOI:** 10.3390/polym16243610

**Published:** 2024-12-23

**Authors:** Xin Li, Lingchen Mao, Weiqian Li, Han Wu, Suyang Dai, Rui Xiao, Jiayi Huang, Guodong Liu, Keda Yang, Wensheng Bu, Ni Jiang, Zhihua Gan, Zhenbo Ning

**Affiliations:** 1 State Key Laboratory of Organic-Inorganic Composites, Beijing Laboratory of Biomedical Materials, College of Life Science and Technology, Beijing University of Chemical Technology, Beijing 100029, China; 2023210962@buct.edu.cn (X.L.); 2024400389@mail.buct.edu.cn (L.M.); weiqian_li@126.com (W.L.); wuhan@buct.edu.cn (H.W.); 2021400290@buct.edu.cn (S.D.); puzzleclock9393@gmail.com (R.X.); 19313164326@163.com (J.H.); jiangni@mail.buct.edu.cn (N.J.); zhgan@mail.buct.edu.cn (Z.G.); 2 IMElK Technology Development Co., Ltd., Beijing 100010, China; 3 Key Laboratory of Artificial Organs and Computational Medicine in Zhejiang Province, Shulan International Medical College, Zhejiang Shuren University, Hangzhou 310015, China; 4 CAS Key Laboratory of Engineering Plastics, Institute of Chemistry, Chinese Academy of Sciences (CAS), Beijing 100190, China; wenshengbu@iccas.ac.cn

**Keywords:** polyurethane, shape memory, polyglycolide, polytetrahydrofuran

## Abstract

A series of polyurethanes (PU-GT) were prepared using polyglycolide-block-polytetrahydrofuran-block-polyglycolide (PGA-PTHF-PGA), polytetrahydrofuran homopolymer (PTHF), glycerol, and hexamethylene diisocyanate (HDI) by a one-pot synthesis method. The non-isothermal crystallization and subsequent heating curves showed that the PTHF component in these polyurethanes could crystallize in a temperature range of −11.5~2.6 °C during the cooling process, and the melting temperatures of PTHF crystallites were in the range of 24.0~26.9 °C. The WAXD results implied that a small content of PGA could crystallize in the membranes of PU-GT polyurethanes. And compared with PU-GT-100, which did not contain the PGA-PTHF-PGA block polymer, other PU-GT polyurethanes showed excellent mechanical properties because of the existence of the PGA component. Moreover, these polyurethanes had temperature-responsive shape memory properties due to the PTHF crystallites. The temporary shape could be fixed at −20 °C and recovered to the permanent shape at 37 °C. We conducted two kinds of conceptual experiments using PU-GT-50 polyurethane, which showed its great potential for medical applications in vascular and wound repair.

## 1. Introduction

Shape memory polymers (SMPs) can change their shapes in a pre-designed manner in response to specific external stimuli [1,2,3,4]; when heated above a transition temperature (*T*_trans_), an SMP can be programmed via a mechanical stimulus and set into a secondary shape during cooling [5]. With this feature, SMPs have a good application prospect in biomedical devices, flexible electronic devices, intelligent textiles, and so on [6]. Common SMPs include shape memory epoxy resin [7], polystyrene-based polymer [8], polynorbornene [9], polyacrylamides [10], etc. Among them, polyurethane (PU) stands out for its unique advantages, as its chemical structure can be modified extensively to achieve a wide range of properties [11]. At the same time, PU always shows excellent mechanical properties, which provides favorable conditions for the implementation of shape memory. It is well established that PU is composed of both hard and soft segments. In PU with shape memory properties, the soft segments not only impart plasticity to the PU materials but also act as the switching phase, which enables the shape memory behavior of the PU [12]. Due to the polar incompatibility between the soft and hard segment structures, they disperse and aggregate into different micro-regions to form a phase-separated structure. The segments of the soft-segment phase exhibit softening and hardening behavior with temperature changes and are therefore called the “reversible phase”. This reversible phase promotes changes within the polymer. On the other hand, the hard-segment phase has dipole–dipole solid interactions and hydrogen bonds, which allow it to maintain its structure unaffected by temperature changes. This “fixed phase” enables the polymer to remember its initial shape and ensures that it returns to its original state during shape recovery. The hard segments include polyisocyanates and chain extenders, while the soft segments mainly consist of long-chain polyesters, polyethers, or polyolefins [13]. Biodegradable polyesters such as polylactic acid (PLA) and poly(ε-caprolactone) (PCL) are widely used as soft segments in PU and have attracted increasing interest because of their good biocompatibility and biodegradability, which means that they have great potential for biomedical applications [14,15].

As the simplest aliphatic polyester, polyglycolide (PGA) has excellent mechanical properties, good barrier property, superior heat resistance, and rapid degradation rate [16]. However, the poor solubility in most organic solvents and the low toughness of PGA greatly limit its applications. Copolymerizing it with other flexible polymers is an effective way to overcome these problems. Polytetrahydrofuran (PTHF) was a biocompatible polymer with good flexibility and a low melting point (~30 °C), and it has been used for the preparation of polyester-based and body temperature-responsive SMPs [17]. For example, Ren et al. [18] synthesized a four-armed poly(ε-caprolactone)-polytetrahydrofuran (PCL-PTHF) copolymer with good toughness and shape memory properties. Yan et al. [19] introduced SiO_2_ nanoparticles into the copolymer of PLA and PTHF, and this material also exhibited good shape memory behavior.

However, only utilizing copolymerization is usually not enough to prepare materials with excellent mechanical properties. Preparation of corresponding polyurethane materials can easily produce materials with improved elongation at break and mechanical stress because of the crosslinking structure. In addition, using block copolymers to synthesize polyurethane is a common method to improve the flexibility of materials [20,21]. Moreover, the microstructure of polyurethane can also be manipulated by using block copolymer as prepolymer, thereby regulating the mechanical properties of the material [22]. Based on the above discussion, this work aims to prepare a novel polyurethane using PGA and PTHF block copolymers as the polymer matrix. First, the block copolymer of PGA and PTHF (PGA-PTHF-PGA) was synthesized by using PTHF as a macro-initiator. Then, it was used to prepare a series of new polyurethanes (PU-GT) with hexamethylene diisocyanate (HDI), PTHF homopolymer, and glycerol. (PU-GT is a crosslinked polyurethane synthesized by using PGA-PTHF-PGA triblock copolymer and PTHF as the polymer diols. Therefore, we used G to represent PGA and T to represent PTHF, and the polyurethane was named PU-GT.) The crystallization behavior of the different polyurethanes were studied to determine their shape memory temperatures. We further investigated the mechanical properties and shape memory performance of polyurethanes. Moreover, the PU-GT polyurethane can be used as wound dressing by manipulating the contraction force of the wound site through shape memory behavior. To provide the adhesive ability for the polyurethane membrane, a layer of hydrogel prepared from sodium alginate (SA), acrylamide (AM), and *N*,*N*′-methylenebis (acrylamide) (MBA) was crosslinked and fixed on the surface of polyurethane to form a double-layer structure (PU-GT/SA). Finally, we demonstrated the potential applications of this polyurethane in the biomedical field.

## 2. Experimental Section

**Materials.** Glycolide (GA, >99%) was purchased from Corbion PURAC (Amsterdam, The Netherlands) and recrystallized from anhydrous ethyl acetate before use. Polytetrahydrofuran homopolymer (PTHF, *M*_n_ = 2.9 × 10^3^ g/mol) was purchased from Macklin (Shanghai, China) and was dried in a vacuum oven at 80 °C for 2 h before use. Toluene (AR grade, Sinopharm Chemical Reagent, Shanghai, China) was distilled in the presence of metallic sodium and benzophenone. Stannous octoate (Sn(Oct)_2_) was dissolved in dehydrated toluene before use. Glycerol and hexamethylene diisocyanate (HDI) were purchased from Sigma-Aldrich (St. Louis, MO, USA). Acrylamide (AM, 99%) was purchased from Aladdin Reagent Co., Ltd. (Shanghai, China). Sodium alginate (SA, 200−500 Pa s) was purchased from Rhawn (Shanghai, China). *N*,*N*′-methylenebis(acrylamide) (MBA) was purchased from Macklin (Shanghai, China). 1,4-dioxane and *N*,*N*-dimethylformamide (DMF) (99.9%, dry with molecular sieves, water ≤ 30 ppm) were purchased from InnoChem (Beijing, China). All other chemicals were used as received.

**Synthesis of PGA-PTHF-PGA Triblock Copolymers.** PGA-PTHF-PGA triblock copolymers were synthesized by ring-opening polymerization (ROP), in which the PTHF was used as the macro-initiator. The synthesis procedure was as follows: A dried Schlenk flask was degassed and purged with nitrogen three times. Then, predetermined amounts of PTHF (5.8 g) were added and vacuum dried for 2 h at 110 °C in an oil bath. After that, GA (2 g), toluene solution of Sn(Oct)_2_ (3 mol/L, 27 μL), and DMF (13 mL) were added to the flask sequentially under nitrogen. After sealing the Schlenk flask, it was placed in an oil bath at 110 °C and allowed to react for 48 h. Finally, the mixture was isolated by precipitation in excess methanol. The product was dried at 40 °C in a vacuum oven for 1 week before use.

**Preparation of Polyurethanes.** A dried Schlenk flask was degassed and purged with nitrogen three times. Then, predetermined amounts of the synthesized PGA-PTHF-PGA block copolymer, PTHF homopolymer, glycerol, and 1,4-dioxane were added into the flask under nitrogen. In the PU-GT crosslinked polyurethane system, glycerol is used as a crosslinking agent during the synthesis process, causing the polyurethane to form Y-shaped chemical crosslinking points. Then, the mixture was completely dissolved at 80 °C and cooled to room temperature. HDI and Sn(Oct)_2_ were added, and the mixture was stirred for 5 min. After that, it was transferred to a polytetrafluoroethylene mold. The reaction was continued in an oven at 80 °C for 8 h and then vacuum-dried for 48 h to obtain a polyurethane (PU) film. The content of each material mentioned in this step is given in Table 1.

**Preparation of double-layer composite material.** PU-GT/hydrogel composite materials were prepared for a simulated experiment on wound dressing applications. The preparation method is as follows. First, a hydrogel pre-polymer solution was prepared using SA (1.5 wt%, 10 mL), AM (2 g), and MBA (4.4 g). Subsequently, a certain amount of ammonium persulfate and tetramethylethylenediamine were added to the solution, mixed evenly, and quickly poured into a mold. Subsequently, the PU-GT-50 film was treated with UV ozone for 30 min and vacuumed to remove residual O_3_ from the surface. The treated film was then applied to the hydrogel. After being stored in a sealed container at room temperature for 24 h, the PU-GT-50/hydrogel bilayer material (PU-GT-50/SA) was obtained. The reaction scheme of hydrogel is shown in Figure S1.

**Nuclear Magnetic Resonance (NMR).** The ^1^H NMR spectra of the polymerization products were recorded at room temperature with CDCl_3_ as solvent using a Bruker Avance III 400 MHz spectrometer (Ferelden, Switzerland), and the average molecular weights obtained from the ^1^H NMR spectra were calculated according to the following equation [23]:

(1) $$M_{n}=2900+2\times \left[58\times \left(I_{4.83}/I_{4.19}+1\right)+59\right]$$

where 2900 g/mol is the molecular weight of PTHF. *I*_4.83_ refers to the peak (4.83 ppm) integral area of the methylene proton in PGA repeat unit, and *I*_4.19_ refers to the peak (4.19 ppm) integral area of the methylene proton of the PTHF unit closest to the PGA blocks.

**Gel Permeation Chromatography (GPC).** The relative molecular weights of the polymers were measured with a Waters 1515 gel permeation chromatography system. The measurements were carried out at 45 °C with DMF as the eluent and polystyrene as the standard at a flow rate of 1.0 mL/min.

**Fourier Transform Infrared Analysis (FTIR).** FTIR spectra of the samples were obtained by a Perkin-Elmer Spectrum 100 (Waltham, MA, USA) equipped with an attenuated total reflection (ATR) accessory in the wavelength range of 4000 to 400 cm^−1^ with a resolution of 4 cm^−1^ and 16 scans.

**Differential Scanning Calorimetry (DSC).** Thermal analysis was performed using a TA-Q2000 Differential Scanning Calorimeter (New Castle, DE, USA) after calibration with indium and hexatriacontane in a nitrogen atmosphere. Approximately 6 mg of the samples was encapsulated in aluminum trays for DSC measurements. For the analysis of non-isothermal crystallization, the samples were first melted at 230 °C for 3 min to eliminate thermal history, then cooled to −40 °C at 10 °C/min, and then they were reheated to 230 °C at 10 °C/min.

**Wide-Angle X-Ray Diffraction (WAXD).** WAXD patterns of the samples were recorded on a Rigaku 2500VB2+/PC diffractometer (Tokyo, Japan) with nickel-filtered Cu Kα radiation (wavelength λ = 0.154 nm, 40 kV, and 110 mA) in the 2*θ* range from 5° to 40° at a scanning step of 0.02°.

**Tensile Test.** The tensile tests were carried out using a CMT-4204 motorized tensile testing machine (Shenzhen, China). The testing force range of this instrument is 0.4% to 100% of the full scale. We conducted tensile testing according to the ASTM D1708 standard [24], which specifies the preparation and testing methods for miniature dog-bone-shaped specimens. The films were tested by cutting them into dumbbell-shaped samples (20 × 2 × 0.2 mm) through a punching machine. The tensile rate was set at 10 mm/min, and the tests were conducted at room temperature. The tensile strength of all specimens was obtained at the maximum stress of the stress–strain curve before rupture. Each set of experiments was repeated five times, and the average of the test results was calculated to ensure the reproducibility of the data.

**Shape Memory Test.** The shape memory properties of the material were determined by a folding and unfolding test. The angle change of the polymer strip was measured, and the shape fixation (*R*_f_) and shape recovery (*R*_r_) were calculated according to the following equations [25,26]:

(2) $$\mathrm{Extent of shape fixity}\;R_{f}=\frac{\theta_{f}}{\theta_{m}}\times 100\%$$

(3) $$\mathrm{Extent of shape recovery}\;R_{r}=\frac{\theta_{f}-\theta_{r}}{\theta_{f}}\times 100\%$$

The sample was fixed to a specific shape and kept at −20 °C for 3 min, then the maximum angle of deformation (*θ*_m_) was recorded, and the fixed angle (*θ*_f_) was measured at −20 °C after it was removed from the setting mold. Then, it was placed in deionized water at 37 °C for 1 min, and the recovery angle (*θ*_r_) was measured after removal. The fixed and recovered spline were separately photographed, and the angle between the extension lines on both sides of the U-shaped spline was obtained in the software, with a measurement accuracy of 1.0°.

**Adhesiveness Tests.** The adhesive strengths of PU-GT-50/SA on a pig skin sample were determined using an HP-20 pull-and-push dynamometer (Yueqing, China). The PU-GT-50/SA were applied to the substrate surface (the pig skin sample) with a bonding area of 10 mm × 10 mm and then subjected to pulling until failure under ambient conditions. The maximum force was recorded and normalized to the initial bonding area. For all specimens, the average values of at least three samples were reported.

**Cell Culture.** Mouse epithelial-like fibroblasts (L929) were cultured in Dulbecco’s Modified Eagle’s Medium (DMEM) supplemented with 10% fetal bovine serum (FBS) and 1% penicillin–streptomycin. The cells were maintained at 37 °C in a humidified atmosphere containing 5% CO_2_. Upon reaching confluence, the cells were passaged by detaching them from the cell culture dish using 0.25% trypsin.

**Cell Cytotoxicity Test.** The PU-GT samples were cut into small pieces measuring 2 mm × 2 mm × 1 mm and sterilized with ethanol for 2 h. After washing the samples 3 times with phosphate-buffered saline (PBS), each sample was placed into a well of a 96-well plate and cultured with 200 μL of cell culture medium for 24 h to remove any ethanol and PBS that may have remained to prevent it from affecting the results of the experiment. Subsequently, the medium was removed, and an L929 cell suspension with a density of approximately 5 × 10^3^ cells/well in 200 μL of medium was added to each well. At predetermined time intervals, the Cell Counting Kit-8 (CCK8) assay was employed to assess cell viability. The cell medium was removed, and 100 μL of CCK8 was added to each well. After a 1 h incubation, 80 μL of the CCK8 solution in each well was transferred to an empty 96-well plate, and the optical density (OD) was measured by using a Multiskan MK3 microplate reader (Waltham) at a wavelength of 450 nm. Cell viability was calculated by using the following equation [27]:

(4) $$\mathrm{Cell viability}={OD}_{s}/{OD}_{control}\times 100\%$$

where *OD*_s_ represents the absorbance intensity of the cells co-cultured with PU-GT specimens at 450 nm, and *OD*_control_ denotes the absorbance intensity of the cells incubated with only the culture medium.

## 3. Results and Discussions

**Figure 1:**
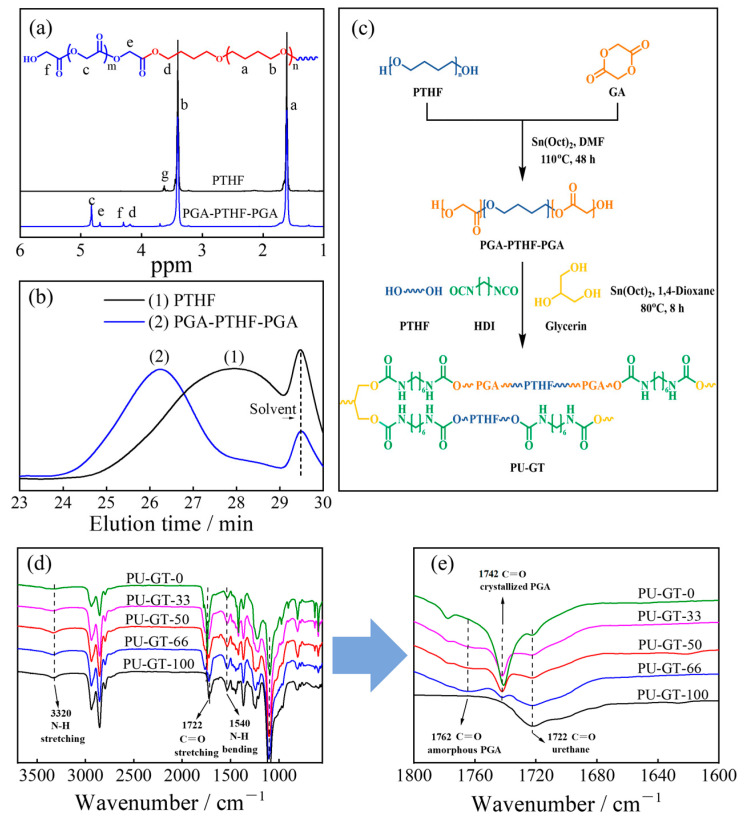
The synthesis of PU-GT polyurethane. (**a**) ^1^H NMR spectra of PTHF and PGA-PTHF-PGA. (**b**) GPC curves of PTHF and PGA-PTHF-PGA. (**c**) Synthetic route. (**d**) FTIR curves (3700–550 cm^−1^) of PU-GT polyurethanes with different compositions. (**e**) FTIR curves (1800–1600 cm^−1^).

**Preparation of PU-GT polyurethanes with Different Compositions.** Considering the poor solubility of PGA in common organic solvents and the need for uniform distribution of PGA within the PU structure, a PGA-PTHF-PGA block polymer was first synthesized via ROP to facilitate the preparation of the PU materials. Figure 1a shows the ^1^H NMR spectrum of the PGA-PTHF-PGA block polymer. The signals at 1.61 ppm (a) and 3.40 ppm (b) were attributed to the different methylene protons in the repeat unit (-CH_2_CH_2_CH_2_CH_2_O-) of PTHF [23], while the signal at 4.83 ppm (c) was attributed to the methylene protons in the repeat unit (-OCH_2_CO-) of PGA [28]. The proton peak at 4.19 ppm (d) was attributed to the methylene protons of PTHF next to the PGA block, while the signal at 4.68 ppm (e) was assigned to the methylene protons of PGA next to the PTHF block. The peak at 4.3 ppm was corresponding to the protons of the CH_2_ (f) in the end GA unit in the block polymer. It should be noted that the signal of 3.62 ppm (g) assigned to the methylene next to the hydroxyl group in PTHF disappeared after the synthesis of the block polymer, which indicated that the hydroxyl groups reacted as the PTHF was the initiator for the ROP of PGA. Figure 1b shows the GPC result of the PGA-PTHF-PGA block copolymer and the PTHF macro-initiator. The elution time of PGA-PTHF-PGA was significantly shorter than that of the PTHF homopolymer, further confirming the successful synthesis of the block copolymer. In the PGA-PTHF-PGA triblock copolymer, the PGA chain segment has a relatively low molecular weight, making the overall molecular weight closer to that of pure PTHF. As we designed, when the two are used together as a polymer diol, the ability of PGA to self-crystallize could improve the material’s strength, while also ensuring a more regular distribution of soft and hard segments in the PU-GT polyurethane system, preventing the loss of mechanical performance. Table S1 shows the characteristics of the synthesized PGA-PTHF-PGA obtained from the GPC and ^1^H NMR results.

The polyurethane was prepared by using the PGA-PTHF-PGA block polymer, PTHF homopolymer with two hydroxyl groups (HO-PTHF-OH), HDI, and glycerol by a one-pot synthesis method, in which PGA-PTHF-PGA and PTHF served as two kinds of polymer diols (Figure 1c), which were the main polymer components in the polyurethanes. Through this method, we synthesized a series of polyurethanes with varying ratios of PTHF homopolymer and PGA-PTHF-PGA triblock copolymer (Table 1). The polyurethane samples were named PU-GT-x, where x represented the molar ratio of PTHF homopolymer in the two kinds of polymer diols used for the synthesis of polyurethane. The weight ratio of PGA (*m*_PGA_) in the polymer diols used for each PU-GT polyurethane is also shown in Table 1. The FTIR spectra of the synthesized PU-GTs are shown in Figure 1d,e. The absorption peak at 3320 cm^−1^ and 1540 cm^−1^ was attributed to N-H stretching and bending vibration of PU-GT, respectively (Figure 1d). The absorption peak at 1722 cm^−1^ was attributed to the stretching vibration of carbonyl (-C=O) of the urethane carbamate [29,30]. The appearance of these peaks means the successful synthesis of PU-GT polyurethane. At the same time, we also found that there was no absorption peak at 2270 cm^−1^ which corresponded to the −NCO groups, indicating that the HDI in the system had reacted completely with the hydroxyl groups [31]. Moreover, the absorption peaks at 1742 cm^−1^ and 1762 cm^−1^ (Figure 1e) were attributed to the carbonyl (C=O) stretching vibration in crystalline and amorphous PGA [32]. These results confirmed the successful preparation of the PU-GT polyurethanes.

**Crystallization Behaviors of PU-GT Polyurethanes.** The crystallization behavior of PU-GT plays an important role in its shape memory properties. As it was shown in Figure 1e, the intensity of the absorption peak at 1742 cm^−1^, which was attributed to the carbonyl (C=O) stretching vibration in PGA crystallites, decreased with the increase in the PTHF homopolymer ratio in PU-GT. This indicates that the PGA chains in the PU-GT could crystallize, and its crystallites increased with the content of the PGA-PHF-PGA block polymer used for the synthesis of polyurethane.

**Figure 2:**
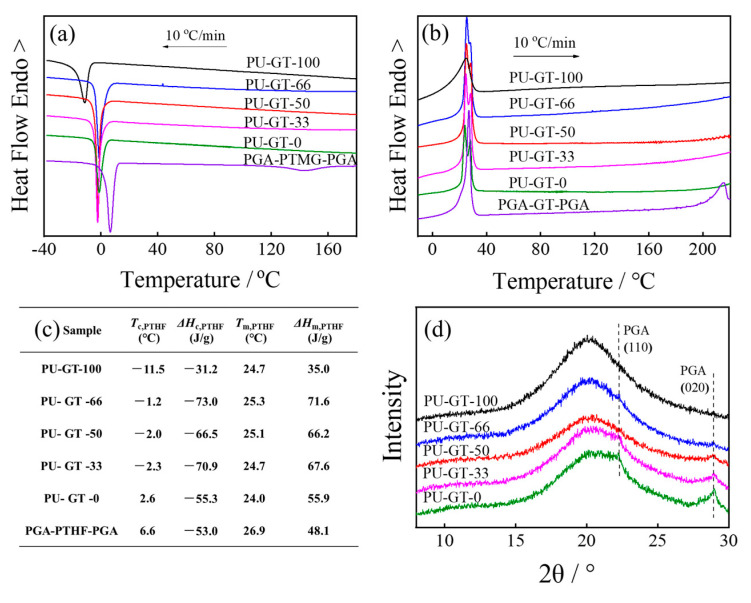
Crystallization behaviors of PU-GT polyurethanes with different compositions. (**a**) DSC curves of non-isothermal crystallization at a rate of 10 °C/min from 230 °C to −40 °C. (**b**) The subsequent heating curves at a rate of 10 °C/min from −40 °C to 230 °C. (**c**) Thermal properties obtained from the non-isothermal DSC analysis; *T*_m, PTHF_ is the melting temperature of PTHF component during the heating process, *T*_c, PTHF_ is the crystallization temperature of PTHF component during the cooling process, *ΔH*_c, PTHF_ is the exothermic enthalpy of PTHF component obtained from non-isothermal DSC analysis, and *ΔH*_m, PTHF_ is the fusion enthalpy of PTHF obtained from the heating process. (**d**) WAXD results of different PU-GT polyurethanes.

The DSC experiments were carried out, and the results are shown in Figure 2. Figure 2a shows the non-isothermal crystallization process of PU-GT with different compositions. For the neat PGA-PTHF-PGA triblock polymer, it showed an obvious crystallization peak at approximately 144.0 °C during the cooling process. This could be attributed to the crystallization of the short PGA chains in the block polymer. However, there was no crystallization peak shown in the cooling curves of the PU-GT because the crystallization of PGA chains was limited significantly by the crosslinking structure of polyurethane. In the temperature range of −30 °C to 15 °C, an exothermic peak emerged in each sample, which was due to the crystallization of PTHF chains in PU-GT [33]. As we can see, the crystallization temperature of PTHF (*T*_c, PTHF_) was about 6.6 °C in PGA-PTHF-PGA, while the *T*_c, PTHF_ decreased to −2.3~2.6 °C for PU-GT containing PGA-PTHF-PGA. PU-GT-100 had the lowest *T*_c, PTHF_ of −11.5 °C. The above results indicate that PTHF in the polyurethane would crystallize at a higher temperature during the cooling process due to the existence of the block polymer. PU-GT-100 was prepared by only PTHF as polymer diol, while other PU-GTs were prepared by both PTHF and PGA-PTHF-PGA as polymer diols, and their *T*_c, PTHF_s would be promoted by the block polymer, so PU-GT-100 showed the lowest crystallization temperature. The increase in the crystallization temperature means the temporary shape fixing temperature of polyurethane can be manipulated, approaching a temperature more suitable for the human body.

Figure 2b shows the subsequent heating curves of all the samples after the non-isothermal crystallization process. The melting peak of PGA crystallites at 215.2 °C existed only in the curve of neat PGA-PTHF-PGA triblock polymer, and it was also difficult to observe this melting peak of PGA for PU-GTs because of the low content PGA in polyurethane. The endothermic peak at 26.9 °C (Figure 2b,c) in PGA-PTHF-PGA was attributed to the melting of PTHF crystallites, so the endothermic peaks at about 25 °C (Figure 2b,c) in PU-GTs were also attributed to the melting of PTHF crystallites. And these results were similar to the cooling process. Figure 2d showed the WAXD results of the PU-GT polyurethane films. The diffraction patterns for PU-GT-66, PU-GT-50, PU-GT-33, and PU-GT-0 reveal minor diffraction peaks at 2*θ* = 22.2° and 28.9°, and these peaks corresponded to the (110) and (020) crystallographic planes of the *α*-form of PGA crystal [34,35]. This indicates that a small content of PGA crystal existed in PU-GT films. Moreover, the intensities of the peak at 28.9° increased with the content of PGA-PTHF-PGA in PU-GT. No diffraction peak of PTHF could be observed, which confirmed that the PTHF chains in the polyurethanes were in an amorphous state at a room temperature of about 25 °C (as shown in Figure 2c).

**Figure 3:**
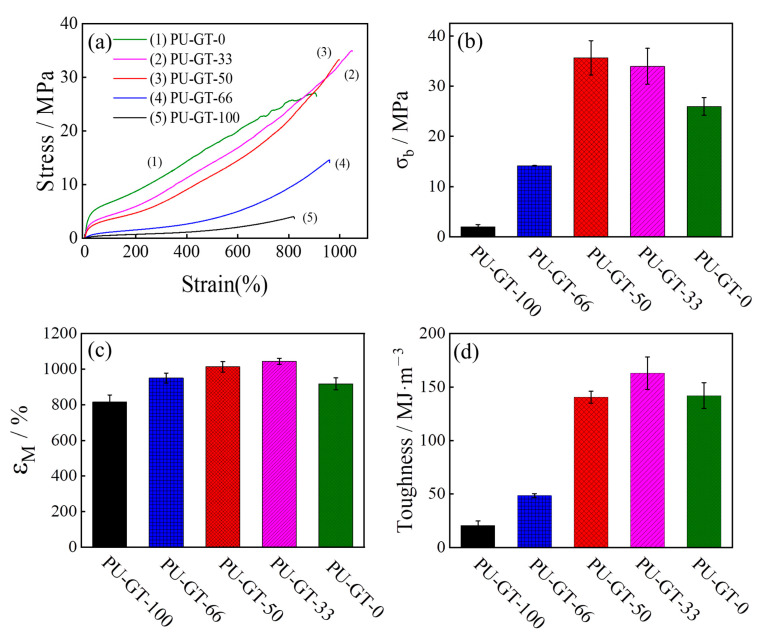
The tensile properties of PU-GT polyurethanes with different compositions. (**a**) Representative strain–stress curves, (**b**) tensile breaking strength, (**c**) elongation at break, and (**d**) toughness. Each experiment was repeated five times, and (**a**) was plotted from the most representative results. The average of the five sets of test results was calculated to obtain (**b**–**d**), and the error bars indicate the standard deviation of each set of data from the average.

**Mechanical Properties of PU-GT Polyurethanes.** The tensile properties of the PU-GT films with different compositions are shown in Figure 3. It should be noted that the tensile strength of PU-GT without the PGA-PTHF-PGA triblock copolymer (PU-GT-100) was the lowest (Figure 3a), and its tensile strength at break was 2.0 ± 0.5 MPa (Figure 3b). However, other PU-GT showed much higher values, which indicated that the introduction of PGA offered superior mechanical properties to PU-GT. When the weight ratio of PTHF homopolymer decreased to 50% (PU-GT-50), the ultimate strength increased to 35.6 ± 3.4 MPa, which was the highest in all the synthesized PU-GTs. The elongation at break (Figure 3c) and toughness (Figure 3d) values followed a similar trend, initially increasing and then decreasing as the PGA-PTHF-PGA content increased. The maximum elongation at break of 1043 ± 18% was observed for the PU-GT-33 film. In PU-GT-100 film without PGA-PTHF-PGA in the polyurethane, the elongation at break decreased to approximately 917 ± 33%. The above results indicated that the mechanical properties of polyurethanes can be adjusted through composition variation, and the excellent mechanical performance of PU-GT served as a foundation for the implementation of shape memory performances.

**Figure 4:**
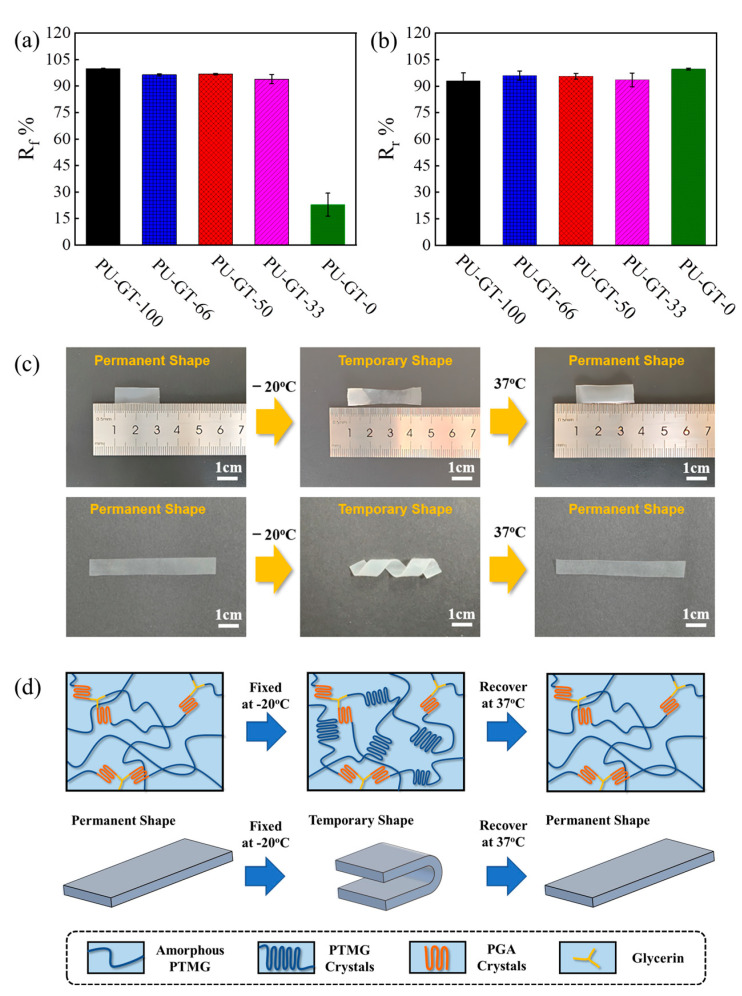
Shape memory behavior of PU-GT polyurethanes. (**a**) The shape fixity ratio (*R*_f_) and (**b**) the shape recovery ratio (*R*_r_). (**c**) Digital photographs showing the shape memory behavior of PU-GT-50. (**d**) Illustration of the shape memory behavior of PU-GT polyurethane.

**Shape Memory Properties of PU-GT Polyurethanes.** The shape memory properties of different PU-GT polyurethanes determined by U-bend model tests are shown in Figure 4. The angle change of the polymer strip is measured, and shape fixation (*R*_f_) and shape recovery (*R*_r_) are calculated according to Equations (2) and (3). The results showed that the shape fixity ratio (*R*_f_) of the PU-GT samples synthesized without the PGA-PTHF-PGA (PU-GT-100) was the highest at 99.8 ± 0.3% (Figure 4a), and its shape recovery ratio (*R*_r_) was 92.9 ± 4.6% (Figure 4b). In contrast, the *R*_f_ of PU-GT-0 (22.8 ± 6.5%) was significantly lower compared to the other *R*_f_ samples because of the lowest content of PTHF in this polyurethane, and the crystallization of PTHF played a role in fixing the temporary shape of the material. In Figure 4c, we chose PU-GT-50 to show the shape memory process of the polyurethane. First, the PU-GT-50 film was softened by placing it in a water bath at 37 °C. Then, it was made into a temporary shape (stretched or crimped) and placed at −20 °C for 3 min to fix the temporary shape. After that, it was transferred into warm water at 37 °C and returned to the original shape within 30s.

In PU-GT films, the PGA crystallites serve as the fixed phase along with chemical crosslinking points during the shape memory process, and the fixed phase maintains the permanent shape of the film at room temperature. While the PTHF component in polyurethane acts as the reversible phase, the temporary shape can be obtained by the crystallization of PTHF, and this phase will be softened when these crystallites are melted and the polyurethane is restored to permanent form (Figure 4d). The above behavior demonstrated that PU-GT-50 possesses a good temperature-responsive shape memory behavior.

**Figure 5:**
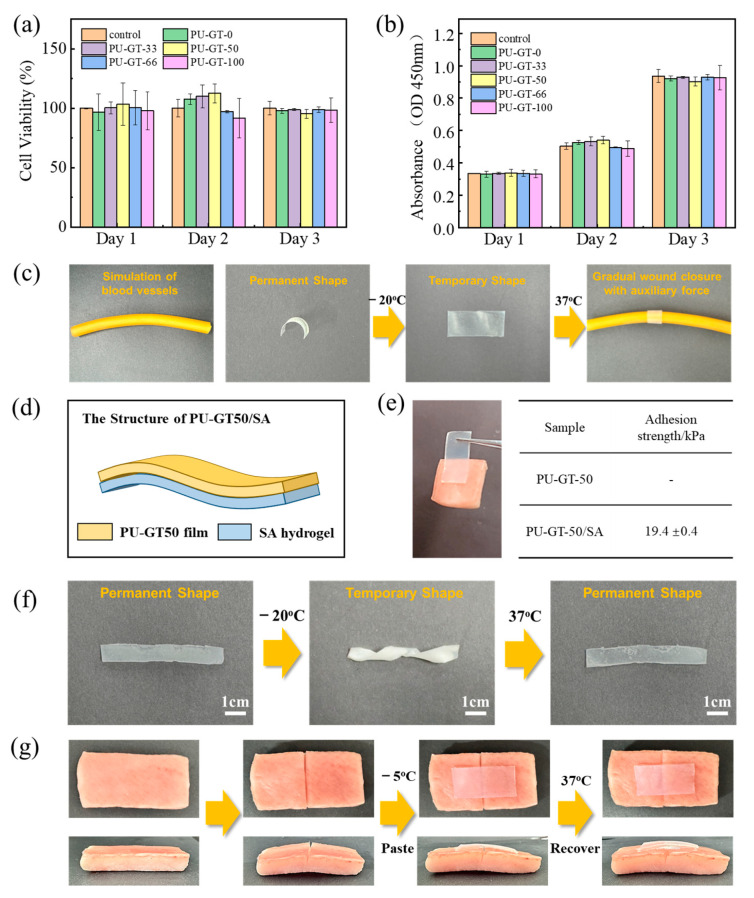
Biocompatibility of the PU-GT polyurethane. (**a**) Cell proliferation and (**b**) viability of L929 osteoblast-like cells cultured with PU-GT polyurethane. (**c**) Illustration of the potential application of PU-GT-50 as vascular repair materials. (**d**) The structure of PU-GT-50/SA. (**e**) Adhesion experiment. (**f**) Shape memory behavior of the double-layer PU-GT polyurethane, showing the permanent shape, the temporary shape after the crystallization at 0 °C, and the restored permanent shape after melting at 37 °C. (**g**) Illustration of the potential application as wound dressing of PU-GT-50/SA.

**Potential Applications of the Shape Memory PU-GT Polyurethane.** To verify the biocompatibility of the synthesized polyurethane, L929 cells were incubated with the PU-GTs, and cell viability was examined using a CCK8 assay. As can be seen in Figure 5a,b, the cell number maintained continuous growth during the culture period of 3 days, and the cell viability is well above 70%, meeting the requirements mentioned in ISO 10993-5 [36]. The viability of L929 cells co-cultured with PU-GT polyurethanes was also close to that of the control group, demonstrating that these samples were noncytotoxic.

PU-GT polyurethane’s excellent mechanical properties, body-temperature-responsive shape memory properties and good cell compatibilities provide great potential for its application in the biomedical field. From Figure 2a,c, we know that the *T*_c, PTHF_ in the PU-GT polyurethane increased from −11.5 °C for PU-GT-100 to −2.0 °C for PU-GT-50 (molar ratio of PTHF as the polymer diol was 50%), which means that the temporary shape of the polyurethane could be fixed at a higher temperature that the human body is more receptive to. And from Figure 3 and Figure 4, we can see that PU-GT-50 has the best mechanical and shape memory performance, so we finally chose this ratio for subsequent experiments. A temporary flat membrane was fixed at −5 °C after the crystallization of the PTHF component in Figure 5c, while the PU-GT-50 membrane was prepared as a curved permanent shape. If the flat film with a fixed flat form was placed on a latex tube at 37 °C (here, we use latex tube at 37 °C to simulate the damaged human blood vessel), due to the melting of PTHF crystals, the shape of the film recovered to the curved tubular shape, thus wrapping around the latex tube and assisting in repairing the vascular tissue. This result mean that PU-GT-50 films had the potential to be used as a material for vascular repair. Furthermore, the PU-GT polyurethane may also have applications in wound dressing, which can enhance the contraction force of the wound site through shape memory behavior. To achieve this function, a layer of SA hydrogel was crosslinked and fixed on the surface of polyurethane to provide the adhesive ability for the polyurethane membrane, and the double-layer structure (PU-GT-50/SA) was constructed (Figure 5d). As shown in Figure 5f, this double-layer polyurethane still had an excellent temperature-responsive property, and the adhesion force of the double-layer membrane on the pig skin was 19.4 ± 0.4 kPa (Figure 5e). A pig skin sample with wounds was utilized for in vitro simulated repair experiments (Figure 5g). Initially, PU-GT-50/SA was stretched at a specific deformation rate, and its temporary shape was fixed at −5 °C. Subsequently, the stretched film adhered to the wound surface. Under the stimulation of body temperature, the shape of the PU-GT-50/SA dressing began to shrink and returned to its initial shape, promoting wound closure and enhancing the mechanical contractile force at the wound site.

## 4. Conclusions

In this study, a PGA-PTHF-PGA triblock copolymer was first synthesized by ring-opening polymerization, and it was used together with PTHF homopolymer, HDI, and glycerol to prepare a novel temperature-responsive shape memory polyurethane. The ^1^H NMR, FTIR, and GPC results indicated the successful synthesis of the block polymer and the PU-GT polyurethanes. The DSC results showed that the presence of the PGA segment promoted the crystallization of PTHF, significantly increasing its crystallization temperature (the crystallization temperature of PU-GT-50 was −2.0 °C). The WAXD results indicated that PGA could still form a crystalline structure within the PU-GT system, thereby providing higher tensile strength and toughness. Additionally, all the polyurethanes exhibited an elongation at break exceeding 800%, meeting the requirements for practical applications. The excellent shape memory behavior of PU-GT-50 was further studied, and two conceptual experiments were carried out based on the temperature-responsive behavior. The polyurethane film showed good biocompatibility in the in vitro toxicity experiments. This material bridges the gap between high mechanical performance and biological compatibility, making it a promising candidate for use in smart medical devices, implants, and other biomedical fields.

## Figures and Tables

**Table 1:** Compositions of different PU-GT polyurethanes.

Sample	PGA-PTHF-PGA (g)	PTHF (g)	Glycerol (mg)	HDI (μL)	*m*_PGA_^1^ (%)
PU-GT-0	1.17	0	18.4	65	25
PU-GT-33	0.78	0.29	18.4	65	17
PU-GT-50	0.59	0.44	18.4	65	15
PU-GT-66	0.39	0.58	18.4	65	10
PU-GT-100	0	0.87	18.4	65	0

^1^ *m*_PGA_: The weight ratio of PGA in PU-GT polyurethane. The ‘X’ in ‘PU-GT-X’ represents the molar ratio of PTHF homopolymer in the two kinds of polymer diols used for the synthesis of polyurethane.

## Data Availability

Due to data protection, detailed data cannot be provided.

## Supplementary Materials

The following supporting information can be downloaded at: https://www.mdpi.com/article/10.3390/polym16243610/s1, Figure S1: The reaction scheme of the hydrogel; Table S1: Characteristics of the synthesized PGA-PTHF-PGA block polymer; Table S2: Tensile properties of the PU-GT films.
