# Supplementary material for: A Novel Polytetrahydrofuran-Based Shape Memory Polyurethane Enhanced by Polyglycolide-Block-Polytetrahydrofuran-Block-Polyglycolide Copolymer
Source: Polymers (Basel). 2024 Dec 23;16(24):3610. doi: 10.3390/polym16243610 (PMC11679042; doi:10.3390/polym16243610)
Supplement: Supplementary file 1 [file polymers-16-03610-s001.zip › polymers-3370356-supplementary.pdf]

Supporting Information

A Novel Polytetrahydrofuran Based Shape Memory Polyurethane Enhanced by Polyglycolide-*block*-Polytetrahydrofuran-*block*-Polyglycolide Copolymer

Xin Li [†,1], *Lingchen Mao* [†,1], *Weiqian Li* [†,1], *Han Wu* [1], *Suyang Dai* [1], *Rui Xiao* [1], *Jiayi Huang* [1], *Guodong Liu* [*,1,4], *Keda Yang* [*,2], *Wensheng Bu* [3], *Ni Jiang* [1], *Zhihua Gan* [1] *and Zhenbo Ning* [*,1]

[1] State Key Laboratory of Organic-Inorganic Composites, Beijing Laboratory of Biomedical Materials, College of Life Science and Technology, Beijing University of Chemical Technology, Beijing 100029, China.

[2] Key Laboratory of Artificial Organs and Computational Medicine in Zhejiang Province, Shulan International Medical College, Zhejiang Shuren University, Hangzhou 310015, China

[3] CAS Key Laboratory of Engineering Plastics, Institute of Chemistry, Chinese Academy of Sciences (CAS), Beijing 100190, China

[4] IMElK Technology Development Co., LTD, Beijing 100010, China

E-mail: liuguodong@imeik.com, kdyang@zjsru.edu.cn, zbning@mail.buct.edu.cn

[†] These authors contributed equally to this work.

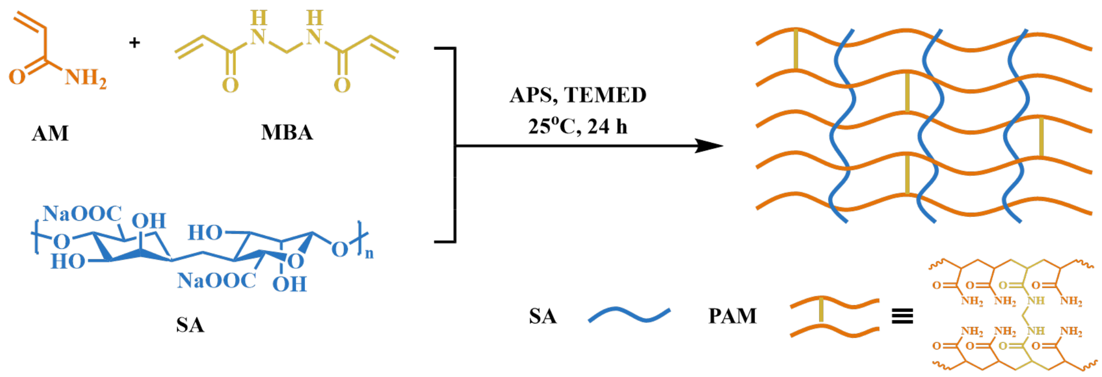

Figure S1. The reaction scheme of the hydrogel

Table S1. Characteristics of the synthesized PGA-PTHF-PGA block polymer.

Sample	M_n [a]	M_w [a]	M_n [b]	PDI [a]
PTHF	1400	1700	2900	1.21
PGA-PTHF-PGA	2900	3500	3800	1.23

[a] Number-averaged molecular weight (M_n), weight-averaged molecular weight (M_w) and molecular weight distribution (PDI) were measured by GPC.

[b] Calculated from ^{1}H NMR results.

Table S2. Tensile properties of the PU-GT films.

Sample	σ_b[a] / MPa	ε_m[b] / %	Toughness[c] / MJ m^{-3}
PU-GT-0	26.0 ± 1.7	917.5 ± 33.4	141.9 ± 12.0
PU-GT-33	34.0 ± 3.6	1043.6 ± 17.7	163.0 ± 15.2
PU-GT-50	35.6 ± 3.4	1012.9 ± 29.0	140.5 ± 5.7
PU-GT-66	14.1 ± 0.1	950.2 ± 27.7	48.6 ± 1.8
PU-GT-100	2.0 ± 0.5	814.7 ± 40.2	20.7 ± 4.2

[a] σ_b was the tensile strength of the samples.

[b] ε_m was the elongation at break of the samples.

[c] The toughness of the samples were derived from integration their stress-strain curves.
